# Supplementary material for: Convergent evolution of SARS-CoV-2 XBB lineages on receptor-binding domain 455–456 synergistically enhances antibody evasion and ACE2 binding
Source: PLoS Pathog. 2023 Dec 20;19(12):e1011868. doi: 10.1371/journal.ppat.1011868 (PMC10766189; doi:10.1371/journal.ppat.1011868)
Supplement: S1 Fig — 50% neutralization titers against SARS-CoV-2 variants of convalescent plasma from individuals who received triple doses of CoronaVac and breakthrough-infected by BA.5 or BF.7 (66 samples). VSV-based pseudoviruses are used. Statistical significances and geometric mean titer (GMT) fold-changes are labeled in comparison with neutralization against XBB.1.5+F456L (the first line) and D614G (the second line). Two-tailed Wilcoxon signed-rank tests of paired samples are used. *, p<0.05; **, p<0.01; ***, p<0.001; ****, p<0.0001; NS, not significant (p>0.05). (PDF) [file ppat.1011868.s002.pdf]

S1 Fig

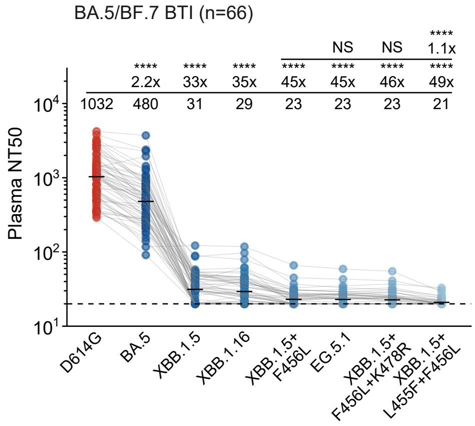

S1 Fig | BA.5 or BF.7 BTI does not elicit strong neutralization against new variants
50% neutralization titers against SARS-CoV-2 variants of convalescent plasma from individuals who received triple doses of CoronaVac and breakthrough-infected by BA.5 or BF.7 (66 samples). VSV-based pseudoviruses are used. Statistical significances and geometric mean titer (GMT) fold-changes are labeled in comparison with neutralization against XBB.1.5+F456L (the first line) and D614G (the second line). Two-tailed Wilcoxon signed-rank tests of paired samples are used. *, $p<0.05$; **, $p<0.01$; ***, $p<0.001$; ****, $p<0.0001$; NS, not significant ($p>0.05$).
