# Supplementary material for: Convergent evolution of SARS-CoV-2 XBB lineages on receptor-binding domain 455–456 synergistically enhances antibody evasion and ACE2 binding
Source: PLoS Pathog. 2023 Dec 20;19(12):e1011868. doi: 10.1371/journal.ppat.1011868 (PMC10766189; doi:10.1371/journal.ppat.1011868)
Supplement: S2 Fig — The antibody names, source cohorts, VDJ genes utilization, somatic hypermutation (SHM) ratio, and CDR lengths are shown in the table. Antibodies with the public IGHV3-53/3-66 heavy chain V genes are marked in blue background. (PDF) [file ppat.1011868.s003.pdf]

S2 Fig

antibody	source	Heavy chain V gene	Heavy chain D gene	Heavy chain J gene	Light chain V gene	Light chain J gene	VH SHM ratio	VL SHM ratio	VH SHM count (nt)	VL SHM count (nt)	CDR-H3 length	CDR-L3 length
GC0325	BA.5 BTI	IGHV4-39	IGHD6-13	IGHJ4	IGLV1-40	IGLJ3	0.030	0.015	11	5	15	12
GC0805	BA.5 BTI	IGHV3-30	IGHD3-16	IGHJ6	IGLV1-51	IGLJ3	0.023	0.033	9	11	21	12
GC1134	BF.7 BTI	IGHV3-33	IGHD3-16	IGHJ4	IGLV3-21	IGLJ2	0.076	0.055	27	18	11	11
GC1140	BF.7 BTI	IGHV4-39	IGHD1-26	IGHJ4	IGLV1-40	IGLJ1	0.024	0.006	9	2	16	12
GC1169	BF.7 BTI	IGHV3-66	IGHD6-6	IGHJ6	IGKV1-33	IGKJ2	0.048	0.003	17	1	13	9
GC1295	BF.7 BTI	IGHV3-66	IGHD1-1	IGHJ6	IGLV3-21	IGLJ3	0.082	0.043	29	14	12	11
GC1360	BF.7 BTI	IGHV3-30	IGHD5-18	IGHJ6	IGKV2-28	IGKJ2	0.003	0.000	1	0	20	9
GC1468	BA.1 BTI + BA.5/BF.7 infection	IGHV3-53	IGHD3-16	IGHJ6	IGKV1-9	IGKJ5	0.080	0.044	28	14	11	9
GC1470	BA.2 BTI + BA.5/BF.7 infection	IGHV3-53	IGHD6-19	IGHJ4	IGKV1-33	IGKJ1	0.080	0.050	28	16	10	9
GC1479	BA.2 BTI + BA.5/BF.7 infection	IGHV3-9	IGHD3-9	IGHJ6	IGKV1-9	IGKJ2	0.056	0.022	22	7	23	9
GC1504	BA.2 BTI + BA.5/BF.7 infection	IGHV3-53	N/A	IGHJ4	IGKV1-33	IGKJ2	0.049	0.019	17	6	10	10
GC1513	BA.1 BTI + BA.5/BF.7 infection	IGHV4-39	IGHD1-26	IGHJ4	IGKV1-NL1	IGKJ4	0.097	0.068	34	22	9	10
GC1518	BA.2 BTI + BA.5/BF.7 infection	IGHV3-53	IGHD4-11	IGHJ6	IGKV1-33	IGKJ3	0.020	0.015	7	5	9	10
GC1538	BA.2 BTI + BA.5/BF.7 infection	IGHV3-33	IGHD2-15	IGHJ6	IGKV1-33	IGKJ4	0.048	0.046	18	15	17	10
GC1540	BA.2 BTI + BA.5/BF.7 infection	IGHV3-66	IGHD3-10	IGHJ6	IGKV1-33	IGKJ3	0.053	0.022	19	7	13	10
GC1544	BA.2 BTI + BA.5/BF.7 infection	IGHV3-11	IGHD5-12	IGHJ4	IGKV1-33	IGKJ5	0.051	0.040	18	13	11	10
GC1552	BA.1 BTI + BA.5/BF.7 infection	IGHV3-33	IGHD3-22	IGHJ6	IGKV1-39	IGKJ5	0.034	0.037	13	12	22	9
GC1556	BA.2 BTI + BA.5/BF.7 infection	IGHV3-66	IGHD2-15	IGHJ4	IGKV1-33	IGKJ2	0.044	0.028	16	9	16	9
GC1621	BA.1 BTI + BA.5/BF.7 infection	IGHV4-39	IGHD1-14	IGHJ4	IGKV1-NL1	IGKJ4	0.048	0.028	17	9	9	10
GC1644	BA.2 BTI + BA.5/BF.7 infection	IGHV3-43D	IGHD3-3	IGHJ6	IGKV1-5	IGKJ1	0.038	0.016	14	5	15	8
GC1678	BA.1 BTI + BA.5/BF.7 infection	IGHV3-30	IGHD5-24	IGHJ6	IGKV4-1	IGKJ2	0.071	0.065	26	22	16	9
GC1687	BA.1 BTI + BA.5/BF.7 infection	IGHV3-30	IGHD3-3	IGHJ6	IGKV2-28	IGKJ4	0.055	0.012	20	4	15	9
GC1688	BA.1 BTI + BA.5/BF.7 infection	IGHV3-30	IGHD3-22	IGHJ6	IGKV2-28	IGKJ2	0.053	0.027	20	9	19	9
GC1699	BA.2 BTI + BA.5/BF.7 infection	IGHV3-9	IGHD5-18	IGHJ3	IGKV2D-29	IGKJ2	0.038	0.024	14	8	16	10
GC1719	BA.2 BTI + BA.5/BF.7 infection	IGHV3-48	IGHD3-16	IGHJ5	IGKV3-11	IGKJ2	0.078	0.022	28	7	13	10
GC1726	BA.1 BTI + BA.5/BF.7 infection	IGHV3-66	IGHD3-22	IGHJ3	IGKV3-20	IGKJ5	0.042	0.044	15	14	12	8
GC1727	BA.2 BTI + BA.5/BF.7 infection	IGHV3-66	IGHD4-17	IGHJ4	IGKV3-20	IGKJ4	0.051	0.055	18	18	11	9
GC1732	BA.1 BTI + BA.5/BF.7 infection	IGHV3-66	IGHD6-19	IGHJ3	IGKV3-20	IGKJ5	0.065	0.040	23	13	12	8
GC1753	BA.2 BTI + BA.5/BF.7 infection	IGHV3-48	IGHD3-10	IGHJ4	IGKV3-20	IGKJ4	0.032	0.022	12	7	16	9
GC1859	BA.2 BTI + BA.5/BF.7 infection	IGHV3-9	IGHD5-24	IGHJ6	IGLV2-23	IGLJ1	0.029	0.027	11	9	18	11
GC1928	BA.1 BTI + BA.5/BF.7 infection	IGHV3-23	IGHD3-3	IGHJ6	IGLV1-51	IGLJ1	0.032	0.012	12	4	20	11
GC1939	BA.1 BTI + BA.5/BF.7 infection	IGHV3-21	IGHD4-17	IGHJ5	IGLV1-40	IGLJ2	0.049	0.027	18	9	15	12
GC2045	BA.2 BTI + BA.5/BF.7 infection	IGHV3-66	IGHD2-21	IGHJ6	IGLV3-21	IGLJ2	0.057	0.037	20	12	11	11
SN1198	BA.2 BTI	IGHV3-53	IGHD5-18	IGHJ4	IGKV1-33	IGKJ3	0.023	0.006	8	2	11	10

S2 Fig | Information of XBB.1.5-neutralizing Class 1 mAbs involved in the study
The antibody names, source cohorts, VDJ genes utilization, somatic hypermutation (SHM) ratio, and CDR lengths are shown in the table. Antibodies with the public IGHV3-53/3-66 heavy chain V genes are marked in blue background.
