# Supplementary material for: Convergent evolution of SARS-CoV-2 XBB lineages on receptor-binding domain 455–456 synergistically enhances antibody evasion and ACE2 binding
Source: PLoS Pathog. 2023 Dec 20;19(12):e1011868. doi: 10.1371/journal.ppat.1011868 (PMC10766189; doi:10.1371/journal.ppat.1011868)
Supplement: S3 Fig — Paired correlation plots show the pairwise relationship between the escape score of L455F/F456L and total escape scores on 455/456. Antibodies are split into two groups according to their total escape scores on 455/456 as described in Fig 3B. (PDF) [file ppat.1011868.s004.pdf]

S3 Fig

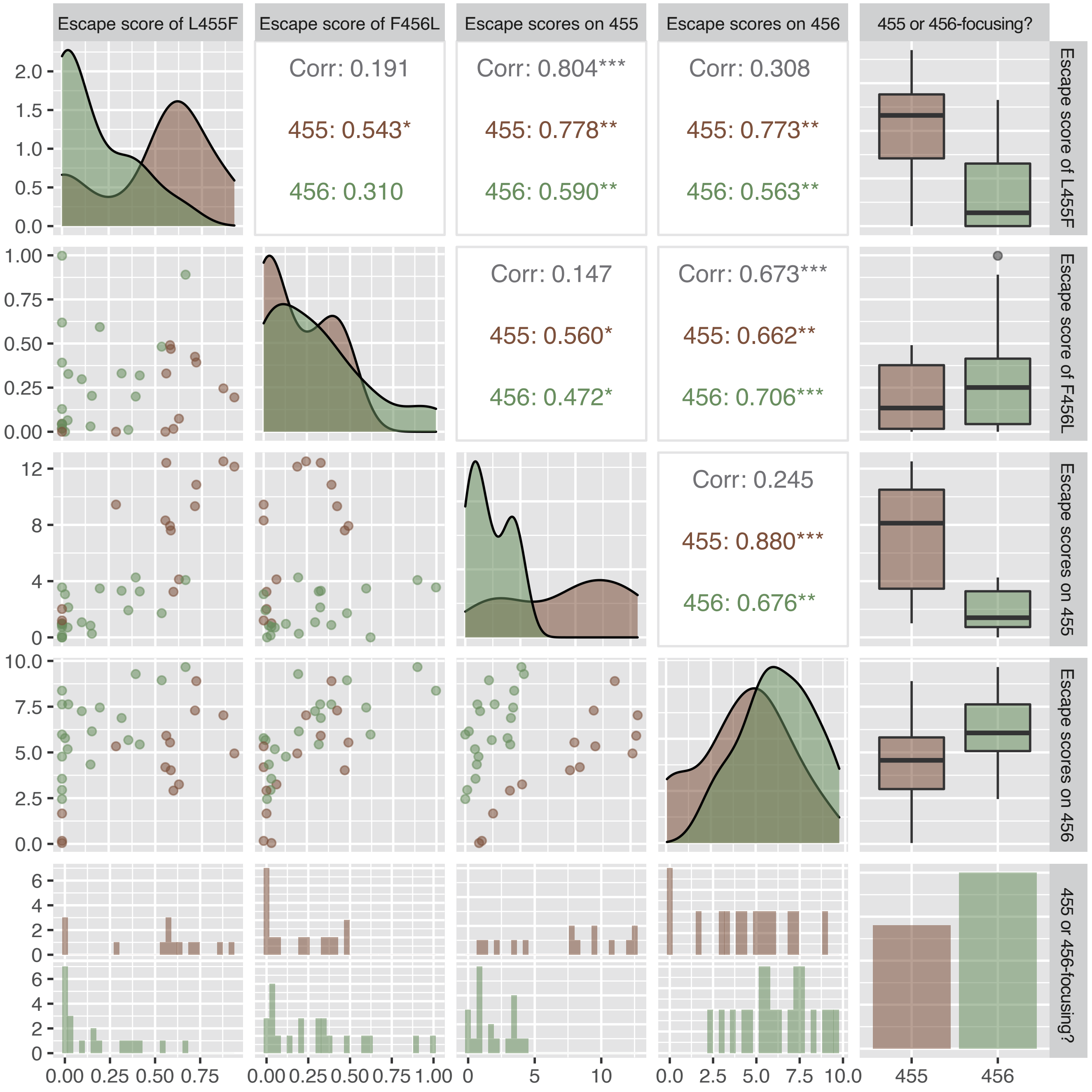

S3 Fig | L455F and F456L show correlated but distinct evasion patterns against mAbs in the DMS dataset
Paired correlation plots show the pairwise relationship between the escape score of L455F/F456L and total escape scores on 455/456. Antibodies are split into two groups according to their total escape scores on 455/456 as described in Fig 3B.
