# Supplementary material for: Convergent evolution of SARS-CoV-2 XBB lineages on receptor-binding domain 455–456 synergistically enhances antibody evasion and ACE2 binding
Source: PLoS Pathog. 2023 Dec 20;19(12):e1011868. doi: 10.1371/journal.ppat.1011868 (PMC10766189; doi:10.1371/journal.ppat.1011868)
Supplement: S4 Fig — (A) IC50 (μg/mL) against D614G, XBB.1.5, XBB.1.5+F456L, XBB.1.5+L455F, and “FLip” (XBB.1.5+L455F+F456L) pseudoviruses using selected XBB.1.5-effective Class 1 monoclonal NAbs. (B) Fold changes of IC50 values compared to IC50 against XBB.1.5 pseudovirus for the Class 1 NAbs. “/” indicates complete escape by the mutant. (PDF) [file ppat.1011868.s005.pdf]

S4 Fig

A

Pseudovirus IC50 (μg/mL)

antibody	D614G	XBB.1.5	XBB.1.5 +F456L	XBB.1.5 +L455F	XBB.1.5 +L455F +F456L
GC0325	>10	0.003	0.062	>10	0.052
GC0805	>10	0.010	0.014	0.011	0.014
GC1134	>10	0.003	>10	0.011	>10
GC1140	>10	0.010	0.016	>10	>10
GC1169	0.010	0.007	0.066	0.058	0.082
GC1295	0.0072	0.033	>10	>10	>10
GC1360	>10	0.075	0.072	>10	>10
GC1468	0.021	0.030	0.811	>10	>10
GC1470	0.0006	0.0013	>10	0.010	>10
GC1479	0.203	0.004	0.009	0.016	0.030
GC1504	0.0050	0.014	0.080	0.186	>10
GC1513	0.0019	0.008	0.016	0.034	0.060
GC1518	0.020	0.030	>10	0.028	>10
GC1538	0.010	0.023	0.281	0.095	>10
GC1540	0.0036	0.002	0.018	0.015	0.023
GC1544	>10	0.001	0.002	0.010	0.033
GC1552	0.012	0.035	>10	0.232	>10
GC1556	0.011	0.029	0.207	>10	>10
GC1621	0.0023	0.020	0.068	0.050	>10
GC1644	>10	0.019	0.016	>10	>10
GC1678	>10	0.059	0.688	0.172	>10
GC1687	>10	0.046	0.050	0.025	>10
GC1688	>10	0.048	0.050	0.265	>10
GC1699	>10	0.032	0.038	0.676	>10
GC1719	>10	0.029	>10	0.028	>10
GC1726	0.0044	0.021	0.146	0.066	>10
GC1727	0.012	0.034	0.192	0.254	>10
GC1732	0.0031	0.052	0.745	>10	>10
GC1753	>10	0.051	0.116	0.082	>10
GC1859	>10	0.037	>10	>10	>10
GC1928	>10	0.0078	0.012	0.017	0.061
GC1939	>10	0.0036	0.584	0.129	>10
GC2045	0.0091	0.048	0.510	>10	>10
SN1198	>10	0.014	>10	>10	>10

B

Pseudovirus IC50 fold change compared to XBB.1.5

antibody	XBB.1.5 +F456L	XBB.1.5 +L455F	XBB.1.5 +L455F +F456L
GC0325	20	/	17
GC0805	1.4	1.1	1.5
GC1134	/	3.3	/
GC1140	1.5	/	/
GC1169	10	8.9	13
GC1295	/	/	/
GC1360	1.0	/	/
GC1468	27	/	/
GC1470	/	8.2	/
GC1479	2.3	3.8	7.4
GC1504	5.9	14	/
GC1513	2.0	4.2	7.5
GC1518	/	0.9	/
GC1538	12	4.1	/
GC1540	8.5	7.2	11
GC1544	1.7	8.5	28
GC1552	/	6.7	/
GC1556	7.1	/	/
GC1621	3.4	2.5	/
GC1644	0.8	/	/
GC1678	12	2.9	/
GC1687	1.1	0.6	/
GC1688	1.0	5.5	/
GC1699	1.2	21	/
GC1719	/	1.0	/
GC1726	6.8	3.1	/
GC1727	5.6	7.4	/
GC1732	14	/	/
GC1753	2.3	1.6	/
GC1859	/	/	/
GC1928	1.5	2.2	7.8
GC1939	162	36	/
GC2045	11	/	/
SN1198	/	/	/

S4 Fig | Neutralization activities of Class 1 NAbs against variant pseudoviruses

(A) IC50 (μg/mL) against D614G, XBB.1.5, XBB.1.5+F456L, XBB.1.5+L455F, and "FLip" (XBB.1.5+L455F+F456L) pseudoviruses using selected XBB.1.5-effective Class 1 monoclonal NAbs.

(B) Fold changes of IC50 values compared to IC50 against XBB.1.5 pseudovirus for the Class 1 NAbs. "/" indicates complete escape by the mutant.
