# Supplementary material for: Convergent evolution of SARS-CoV-2 XBB lineages on receptor-binding domain 455–456 synergistically enhances antibody evasion and ACE2 binding
Source: PLoS Pathog. 2023 Dec 20;19(12):e1011868. doi: 10.1371/journal.ppat.1011868 (PMC10766189; doi:10.1371/journal.ppat.1011868)
Supplement: S5 Fig — Association (A) and dissociation (B) constants of the binding kinetics between XBB variants RBD and hACE2 determined by SPR. Each dot indicates one independent SPR measurement. Geometric mean values are shown as bars. (PDF) [file ppat.1011868.s006.pdf]

S5 Fig

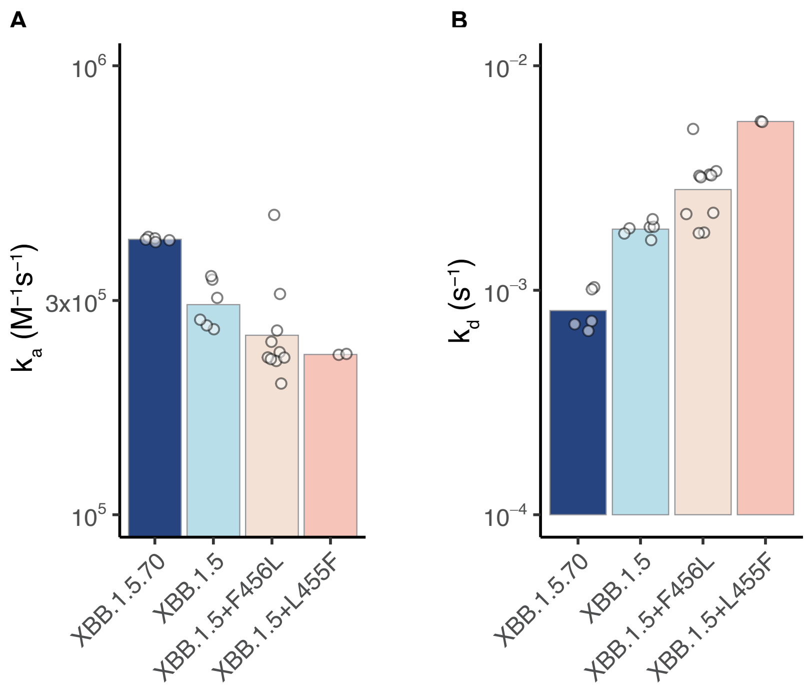

S5 Fig | Kinetic parameters for RBD-ACE2 binding from SPR assays
Association (A) and dissociation (B) constants of the binding kinetics between XBB variants RBD and hACE2 determined by SPR. Each dot indicates one independent SPR measurement. Geometric mean values are shown as bars.
