# Supplementary material for: Convergent evolution of SARS-CoV-2 XBB lineages on receptor-binding domain 455–456 synergistically enhances antibody evasion and ACE2 binding
Source: PLoS Pathog. 2023 Dec 20;19(12):e1011868. doi: 10.1371/journal.ppat.1011868 (PMC10766189; doi:10.1371/journal.ppat.1011868)
Supplement: S6 Fig — Workflow to generate refined structural model of XBB.1.5 Spike, XBB.1.5+F456L (XBB.1.5.10) Spike, XBB.1.5+L455F+F456L (XBB.1.5.70) Spike, XBB.1.5 RBD, XBB.1.5.10 RBD and XBB.1.5.70 RBD in complex of ACE2. (PDF) [file ppat.1011868.s007.pdf]

# S6 Fig

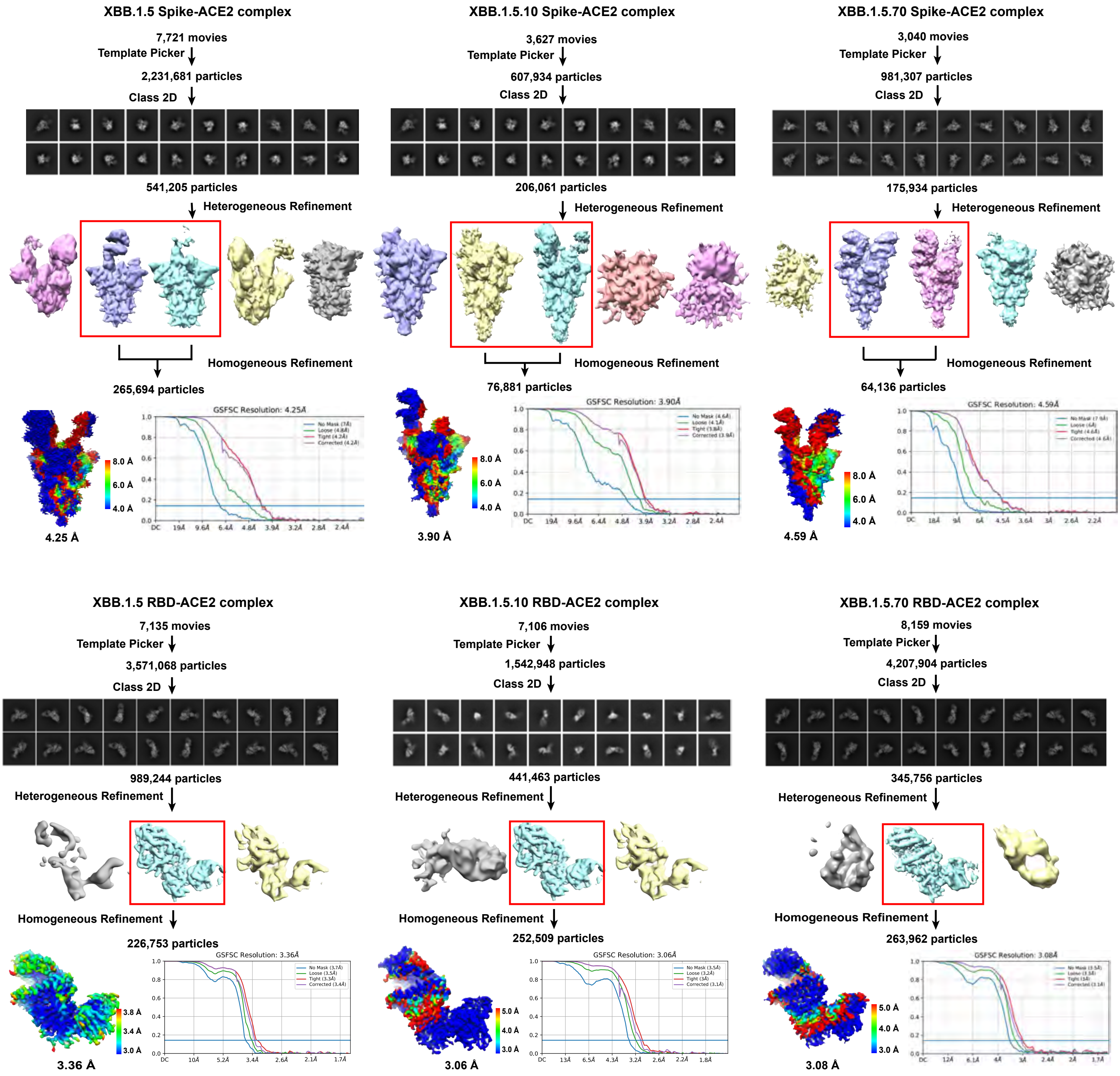


**S6 Fig | Workflow for cryo-EM structural models**

Workflow to generate refined structural model of XBB.1.5 Spike, XBB.1.5+F456L (XBB.1.5.10) Spike, XBB.1.5+L455F+F456L (XBB.1.5.70) Spike, XBB.1.5 RBD, XBB.1.5.10 RBD and XBB.1.5.70 RBD in complex of ACE2.
