# Supplementary material for: Convergent evolution of SARS-CoV-2 XBB lineages on receptor-binding domain 455–456 synergistically enhances antibody evasion and ACE2 binding
Source: PLoS Pathog. 2023 Dec 20;19(12):e1011868. doi: 10.1371/journal.ppat.1011868 (PMC10766189; doi:10.1371/journal.ppat.1011868)
Supplement: S7 Fig — (A) The electron density of XBB.1.5 and XBB.1.5+F456L RBD-ACE2 complex around site 455 and 456 in detail, carbon atoms are shown as sticks, density is shown in mesh, and the color scheme is the same as that in Fig 5C. (B) Superimposition of BA.2 RBD-ACE2 (PDB:7ZF7) and XBB.1.5+F456L RBD-ACE2 complex structure. Hydrogen bonds are shown as yellow dashed lines. Potential steric clash is shown in yellow circles. (PDF) [file ppat.1011868.s008.pdf]

S7 Fig

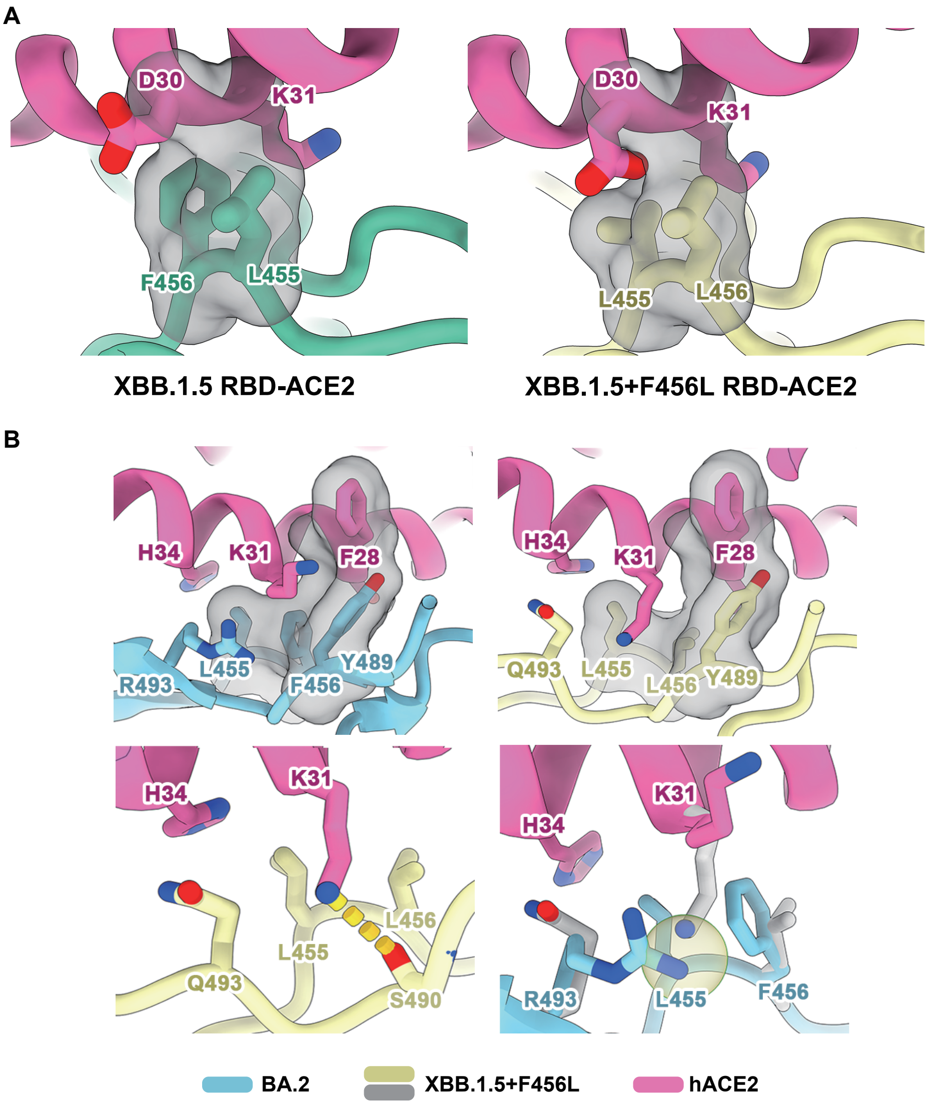

S7 Fig | F456L remodels the interaction between RBD and ACE2

(A) The electron density of XBB.1.5 and XBB.1.5+F456L RBD-ACE2 complex around site 455 and 456 in detail, carbon atoms are shown as sticks, density is shown in mesh, and the color scheme is the same as that in Fig 5C.

(B) Superimposition of BA.2 RBD-ACE2 (PDB:7ZF7) and XBB.1.5+F456L RBD-ACE2 complex structure. Hydrogen bonds are shown as yellow dashed lines. Potential steric clash is shown in yellow circles.
