# Supplementary material for: Convergent evolution of SARS-CoV-2 XBB lineages on receptor-binding domain 455–456 synergistically enhances antibody evasion and ACE2 binding
Source: PLoS Pathog. 2023 Dec 20;19(12):e1011868. doi: 10.1371/journal.ppat.1011868 (PMC10766189; doi:10.1371/journal.ppat.1011868)
Supplement: Attachment — Submitted filename: Response_Letter.docx [file ppat.1011868.s009.docx]

**Responses to the Reviewers’ Questions**

**Reviewer #1:**

L455F mutation has been acquired by multiple independent XBB derivative strains that contain F456L mutation. This study aims to investigate the interaction between L455F and F456L. The authors show that L455F+F456L can better escape both polyclonal plasma samples and monoclonal antibodies. They also show that epistasis exists between L455F and F456L in terms of hACE2 binding. Mechanism of this epistasis is revealed by cryo-EM analysis. Overall, this is a nice case study on a pair of RBD mutation and demonstrates the role of epistasis in the ongoing SARS-CoV-2 evolution.

Response: Thanks for your recognition of our study.

**Major Issues:**

1. Lines 60-61: “However, the lineages with L455F mutation but without F456L did not exhibit any growth advantage [13].” Ref #13 is a bioinformatics paper and does not mention L455F mutation. This statement needs to be supported by additional references or data.

Response: Sorry for the confusion. The Ref.13 is the original paper of CoV-Spectrum (<https://cov-spectrum.org/>), which is one of the most popular SARS-CoV-2 mutation tracking websites that utilizes data from GISAID. As suggested, we added data on “XBB*+S:486P+S:455F+S:456F” (L455F but not F456L) from this website and updated Figure 1C using recent data to support this statement, and modified the text to avoid confusion (Line 61). The number of such sequences is too small to be observable in the stacked bar chart (Figure 1C), which exactly demonstrates that L455F without F456L is deleterious for the virus.

2. Line 97: “L455F and F456L complement each other”. This statement is not true if XBB1.5 + L455F achieves the same or even larger degree of escape compared to XBB1.5 + L455F + F456L. However, XBB1.5 + L455F was not included in the neutralization assay in Figure 2. So there is insufficient evidence to claim that F456L complements L455F.

Response: Thanks for pointing this out. We agree that it is not sufficient to conclude that F456L complements L455F from the plasma neutralization data in Figure 2. In the revised Figure 2, we added the neutralization data against XBB.1.5+L455F, which shows similar degree of evasion compared to XBB.1.5+F456L as expected, demonstrating the complementary effects. In addition, Figure 3B, 3C and S4 show that XBB.1.5+L455F and XBB.1.5+F456L escape different subset of XBB-neutralizing Class 1 antibodies. These mAb-level analyses also support the conclusion that L455F and F456L complement each other to achieve maximal immune escape. The corresponding description has been added to the main text (Line 97).

3. Lines 160-162: “This unique pattern confers more flexible space for Q493 on RBD and the H34 on ACE2, hence enabling insertion of H34 side chain between RBD Q493 and S494, which cannot be realized in XBB.1.5 or XBB.1.5+F456L.” There is no data to support the claim that Q493 cannot adopt the rotamer seen in Figure 5D in XBB.1.5 or XBB.1.5+F456L. This concern can potentially be addressed by performing an analysis similar to Figure 5E.

Response: Thanks for the suggestion. We added two new subfigures in Figure 5E that visualizes the aligned RBD-ACE2 interface in the opposite direction, which shows that the conformation of Q493 in “FLip” will not be favorable in XBB.1.5 or XBB.1.5+F456L RBD, due to the potential clash between L455 and Q493 (Line 168 in the revised manuscript).

**Minor Issues:**

1. Line 110: “Notably, 11 NAbs were not completely escaped by either L455F or F456L …” The authors may want to describe the definition of “completely escaped”. My understanding is that it means no detected neutralization activity at 10 μg/mL.

Response: Yes, “completely escaped” means IC50 > 10 μg/mL in our context, but interestingly, these Class 1 potent NAbs involved in the study are either “completely escaped”, or exhibit an IC50 < 1 μg/mL. Therefore, “not completely escaped” can also be defined as IC50 < 1 μg/mL here. We have added the definition to the revised manuscript (Line 114).

2. Line 150: “both three spike trimers” should be “all three spike trimers”.

Response: Corrected as suggested.

3. Line 155: “F456L mutation does not substantially affect the interactions on the RBD-ACE2 interface, keeping the hydrophobic packing … ” However, F456L should weaken the hydrophobic packing with hACE2 because L is shorter and less bulkier than F.

4. Line 158: “… negligible impact on ACE2-binding affinity of F456L (Figure 5C and S7).” According to Figure 4A, F456L weakened the binding affinity by almost 2-fold, which is a small but not negligible impact. In line 138, the author also mentioned that “F456L also slightly weakens the binding to hACE2”. So the phrase “negligible impact” seem inappropriate. This concern is related to the previous comment.

Response: Thanks for the suggestion. We modified the description to “Although the side chain size of leucine is smaller than phenylalanine, which should slightly weaken the hydrophobic packing, the packing among RBD-L455, RBD-F456, RBD-Y489, and ACE2-F28/D30/K31 is largely kept in L456 mutant, which is in line with the slightly reduced ACE2-binding affinity of F456L.” (Line 161) to emphasize the weakened hydrophobic packing caused by F456L.

5. Line 159: “distinct rotamer pattern”. Please clarify what this term means.

Response: We have changed the description to “distinct side-chain orientation and conformation compared to L455” to improve clarity (Line 166).

6. Lines 217-219: “detailed experimental structural analyses and computational simulation of XBB.1.5 RBD with these two mutations in complex with ACE2 should be necessary in the future.” I am a bit confused here because the structure of XBB.1.5 RBD in complex with ACE2 has already been determined in this study.

Response: Thanks for pointing out this mistake. This sentence was originally in the previous version of our preprint without experimental structural analyses and we forgot to modify it. This has been modified to “detailed analyses of the impacts these two mutations on other RBD background, particular for the variants with distinct antigenicity compared to XBB subvariants, such as BA.2.86, should be carefully evaluated in the future” in the revised manuscript (Line 231).

7. Line 255, the meaning of “evolution flexibility” is unclear.

Response: Specific to the case discussed in this study, the combination of L455F and F456L but not single substitution enhanced ACE2 binding, which allows the virus to accumulate more mutations to achieve higher capability of immune evasion to gain fitness. We changed this term to “possibility of accumulating mutations” to improve clarity.

**Reviewer #2:**

This a well done and timely study explaining the sign epistasis in ACE2 affinity that appears to have promoted the emergence of the L455F / F456L double mutant. The paper nicely shows that these are both antibody escape mutations (partially but not completely redundant in the antibodies they escape), and the reason they probably have emerged so frequently as double mutants (F456L followed by L455F) is that while each mutation individually impairs ACE2 affinity, together they enhance it.

Given the recent evolution of SARS-CoV-2 these are important results. In fact, many of the findings have admirably already been shared by the authors on Twitter to great interest to the relevant scientific community, and this paper nicely fully reports the results in a comprehensive study.

I strongly support publication of this paper, and just have the two minor comments below:

Response: Thanks for your recognition of our study.

The authors could discuss a bit more the role of Q493 (versus R493). Other work (Taylor and Starr, DOI 10.1101/2023.09.11.557279, figure 3D; Dadonaite et al, DOI 10.1101/2023.11.13.566961, figure 1C) have shown that F456L is much more deleterious on the R493 background of BA.2 than Q493 backgrounds (like XBB.1.5). It would appear that the structural biology work presented here offers a partial explanation for that observation, and this could be briefly mentioned.

Response: The suggested issue has been discussed in the Discussion section of the revised manuscript (Line 215). Although the structures presented in this study do not directly explain the observation that F456L is much more deleterious on the R493 background than Q493 background, we show that in the background of Q493 (XBB.1.5), F456L provides a space for insertion of the side chain of K31 into the RBD, which cannot be achieved in the background of R493 (BA.2) due to the steric clash of R493 (Figure S7B), making the co-occurrence of F456L and R493 deleterious.

Figure 3c, the word "effective" is mis-spelled in the legend.

Response: Corrected as suggested.
